# How birds dissipate heat before, during and after flight

**DOI:** 10.1098/rsif.2023.0442

**Published:** 2023-12-13

**Authors:** Agnès Lewden, Charles M. Bishop, Graham N. Askew

**Affiliations:** ^1^ School of Biomedical Sciences, Faculty of Biological Sciences, University of Leeds, Leeds LS2 9JT, UK; ^2^ Univ Brest, CNRS, IRD, Ifremer, LEMAR, IUEM, F-29280 Plouzané, France; ^3^ School of Natural Sciences, Bangor University, Bangor LL57 2DG, UK

**Keywords:** heat balance, endotherms, thermoregulation, heat loss, thermal imaging

## Abstract

Animal flight uses metabolic energy at a higher rate than any other mode of locomotion. A relatively small proportion of the metabolic energy is converted into mechanical power; the remainder is given off as heat. Effective heat dissipation is necessary to avoid hyperthermia. In this study, we measured surface temperatures in lovebirds (*Agapornis personatus*) using infrared thermography and used heat transfer modelling to calculate heat dissipation by convection, radiation and conduction, before, during and after flight. The total non-evaporative rate of heat dissipation in flying birds was 12× higher than before flight and 19× higher than after flight. During flight, heat was largely dissipated by forced convection, via the exposed ventral wing areas, resulting in lower surface temperatures compared with birds at rest. When perched, both before and after exercise, the head and trunk were the main areas involved in dissipating heat. The surface temperature of the legs increased with flight duration and remained high on landing, suggesting that there was an increase in the flow of warmer blood to this region during and after flight. The methodology developed in this study to investigate how birds thermoregulate during flight could be used in future studies to assess the impact of climate change on the behavioural ecology of birds, particularly those species undertaking migratory flights.

## 1. Introduction

In many endotherms, the regulation of heat exchange with the environment allows them to maintain a stable high body temperature. Increased heat production and retention serve to avoid hypothermia at ambient temperatures below the lower critical temperature, while at ambient temperatures above the upper critical temperature and/or during intense exercise, heat dissipation serves to avoid hyperthermia. The heat dissipation limitation theory [1] hypothesizes that constraints on the maximal capacity to dissipate body heat sets a limit on an animal's energetic expenditure (especially during locomotion) to avoid hyperthermia. This theory is supported by the reduction in the metabolic rate in warmer conditions in birds living in arid environments (e.g. [2,3]) and hot acclimatized birds [4].

During flight, birds exhibit the highest rate of metabolic energy expenditure compared with other modes of locomotion [5,6] with heat production 10–19× higher than at rest [7,8]. Heat dissipation areas (HDAs) or ‘thermal windows’, are areas of the body where the surface temperature (*T*_s_) is elevated well above ambient temperature (*T*_a_) (e.g. *T*_s_ − *T*_a_ = 5–8°C in hummingbirds; [9]) and other areas of the body. Three main HDAs have been identified during flight in birds: (i) the head, in particular the unfeathered hot spot eye area, (ii) the feet, and (iii) the proximal wing with the hot spot around the shoulder area [9–11]. In addition, it appears that in species with relatively large bills, the bill is an important HDA after flight, accounting for 35–60% of total heat exchange in toco toucans (*Ramphastos toco*; [12]), 10–18% in tufted puffins (*Fratercula cirrhata*; [13]) and 1.4–19.9% in southern yellow-billed hornbills (*Tockus leucomelas*; [14]). The relative importance of different HDAs in heat dissipation varies. For example, in flying European starling (*Sturnus vulgaris*), 55% of the overall heat loss was dissipated by the wing with 19% dissipated by the shoulder area hot spot [10]. In addition, heat dissipation varies with flight speed. During flight, overall metabolic rate typically exhibits a U-shaped relationship with speed [15,16]. The overall metabolic rate is equal to the sum of the rate of heat production and mechanical power. Since mechanical power also exhibits a U-shaped relationship with speed [16,17], it is, therefore, expected that the rate of heat loss will also follow a similar U-shaped relationship with flight speed. Heat dissipation during flight has only been quantified in European starling where, contrary to the expectation of U-shaped relationship, heat transfer increased linearly with flight speed, between 6 and 14 m s^−1^ [15]. In flying calliope hummingbirds (*Selasphorus calliope*), the temperature of the shoulder region shows a U-shaped relationship with speed [9], whereas the eyes, feet and the mean body surface temperatures decreased with increasing flight speed [18]. However, since heat loss from a body region is dependent on several factors, including its surface area, air speed and the difference between *T*_s_ and *T*_a_, the relative importance of each HDA in dissipating heat at rest, during and after flight, is currently unclear, having never been quantified.

The aim of the study was to quantify regional variation in *T*_s_ and to calculate whole-body and regional heat dissipation before, during and after flight in lovebirds (*Agapornis personatus*; [19]). We hypothesized that during flight, heat dissipation would increase compared with rest due primarily to the need to dissipate the heat generated by the locomotory muscles as they convert metabolic energy into mechanical work, and the relatively low efficiency of this process (e.g. [16]). It was also hypothesized that the elevated metabolism that occurs following exercise [20] may result in increased heat dissipation immediately following flight, but that this would decrease to pre-flight levels over time. Moreover, the change in the bird's posture, with closed wings after landing [12], may impact upon the relative importance of the HDAs in perched birds. We additionally investigated the effects of the preceding flight duration on *T*_s_ and heat dissipation with the hypothesis that birds that performed relatively longer flights, or flights at more energetically demanding higher or lower speeds, would have higher post-flight *T*_s_ and heat loss.

## 2. Material and methods

Between May and June 2018, seven masked lovebirds (body mass 50.17 ± 0.21 g; mean ± standard error (s.e.)) were trained to fly in an open circuit Eiffel-pattern wind tunnel with a working section with dimensions 0.52 × 0.52 × 1.00 m (width × height × length), 5 days per week at the University of Leeds (Leeds, West Yorkshire, UK; 53°48′ N, 1°33′ W). Airspeed was controlled by setting the speed of the fan; the relationship between fan speed and airspeed was determined on the day and prior to data collection using a Pitot-static probe and differential pressure transmitter (FCO332, Furness Controls, Bexhill-on-Sea, East Sussex, UK). Upstream and downstream of the bird a series of vertical, nylon cord restricted the bird to the working section of the wind tunnel. The RMS turbulence in the centre of the working section was less than 0.35% [21]. Birds were maintained in an indoor aviary with access to water, food and cuttlefish ‘bones’ ad libitum. Birds were flown at up to two randomly selected speeds per day (selected from 5, 6, 7, 8, 9, 10, 11, 12, 13, 14 and 15 m s^−1^ which covered speeds both below and above the minimum power speed (A Lewden, CM Bishop and GN Askew 2018, unpublished data)), for a mean duration of 179 ± 9 s (± s.e. with a minimum flight duration of 144 s and a maximum flight duration of 547 s). Birds performed between 2 and 16 flights at different speeds with a mean of 11 flights per bird and there were no differences in flight speeds between birds (ANOVA; *p* = 0.791) and no individual flew longer than the other (ANOVA; *p* = 0.115).

Birds were recorded continuously throughout these flights from the initial period when they were sitting on a wooden perch in the working section of the wind tunnel before flight (hereafter *pre-flight*), during the flight (hereafter *flight*) and once they had landed and were once more sitting on the perch in the working section of the wind tunnel (hereafter *post-flight*) using a thermal camera (FLIR A65 with a 13 mm lens; accuracy ± 5% of reading; FLIR Systems AB, Sweden). The thermal camera was calibrated using a blackbody source (model 989, Isothermal Technology Ltd, Southport, Merseyside, UK) at a distance of 0.4 m, allowing for the correction of non-uniformity across the field of view. The wind tunnel was switched on and off immediately before and after the *flight*, respectively. *T*_s_ was measured on perched birds in still air during a period of 14 ± 2 s (mean ± s.e.) immediately preceding the flight (i.e. *pre-flight*) and on perched birds in still air immediately after the end of the flight (i.e. *post-flight*) during a period of 39 ± 4 s (mean ± s.e.). Relative humidity (mean of 47.36 ± 0.20% (± s.e.)) and ambient temperature (mean of 23.69 ± 0.02°C (± s.e.); range 23–24.5°C) were recorded.

Thermal videos of the bird were recorded in the lateral view through a hole cut in the side of the wind tunnel at a constant distance (0.4 m) between the thermal imaging camera and the bird. Thermal images were analysed using FLIR Tools software (v. 6.4, FLIR systems, Wilsonville, Oregon, USA). Images were selected where the bird was sharply focused and where its lateral view was parallel to the recording plane of the thermal imaging camera. In the analysis software, emissivity was set to 0.95 [10] and distance to the subject to 0.4 m; *T*_a_ and relative humidity were set accordingly to the values recorded on the day of the experiment. At least two images per bird were selected in *pre-flight* before each flying speed with a mean of 3.4 ± 0.5 images and a maximum of 13 images.

At least one image per speed per bird was selected during *flight* and on six individuals in *post-flight*, with a mean of 3.9 ± 0.3 images and a maximum of nine images selected in *flight* and a mean of 4.7 ± 0.7 and a maximum of 16 images in *post-flight*.

**Figure 1.**
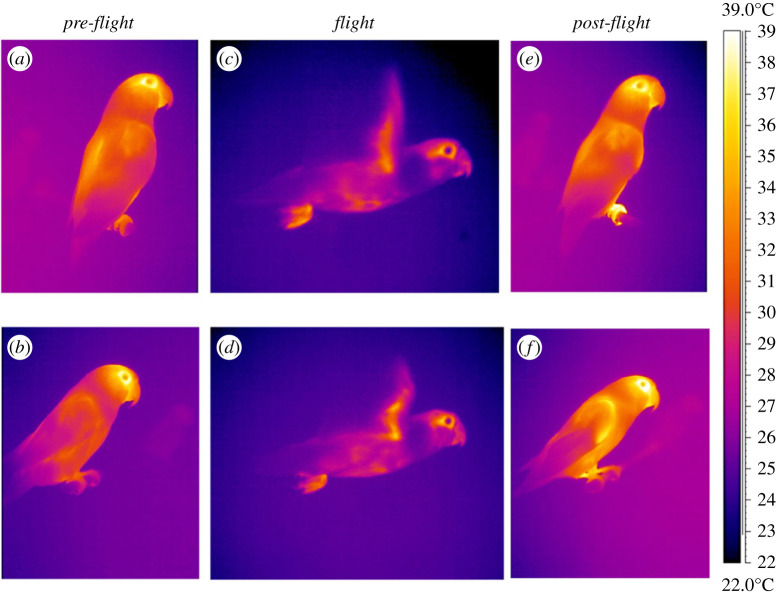
Thermal images of two different perched lovebirds (*a*–*c*–*e* Individual 1 and *b*–*d*–*f* Individual 2) within the wind tunnel working section in *pre-flight* (*a*,*b*), in *flight* (*c*,*d*) and in *post-flight* (*e*,*f*).

For the images selected of the non-handled bird sitting on the perch, polygons were fit to three body areas and the mean *T*_s_ determined. The body areas were: the head (hereafter *head*) including eye and bill hot spots, the legs (hereafter *legs*) including tarsi and feet, and the trunk and the dorsal surface of the folded wing (hereafter *trunk*) in lateral view including a part of the back, the upper surface of the wing and a part of the ventral area (figure 1). Each body area measured was larger than three times the spot size of 0.68 mm with our thermal camera [22,23]. Body surface areas were measured after the birds were euthanized following another study. For each bird, a set of digital images was taken in upstroke lateral position and included a calibration scale. The calibrated images were then analysed using ImageJ software (US National Institutes of Health) to determine the areas of each region of interest. Mean temperature of the wooden perch was determined using a standardized square measuring the size of the width of lovebird's feet. During *flight*, we used the mean *T*_s_ of similar body areas recorded in a lateral view, at the transition between the upstroke and the downstroke: the *head*, the *trunk* (completely visible during late upstroke), *legs* and the ventral proximal wing area in upstroke (hereafter *wing*; figure 1*c*,*d*), including the axillary region (armpit) and the region of the lower surface of the wing held above *T*_a_ (i.e. excluding the flight feathers). Only surface temperatures of sharply focused body areas were used for thermal imaging.

Convective (*q*_conv_) heat loss (W) was calculated for each body area as follows:

2.1 $$q_{\mathrm{conv}}=h_{c}A(T_{S}+T_{a}),$$

where *h*_c_ is the convective heat transfer coefficient, determined using the *Nusselt* number calculated following: method 1 in [10] for the *wing* (wing represented by a flat plate); following method 2 [10] for the *head* (section represented as a sphere) and *trunk* (section represented as cylinder); and following method 3 [10] for the *legs* (section represented as a cylinder). During the *pre-* and *post-flight* periods when the airflow in the wind tunnel was still, *h*_c_ for free convection was used. During *flight* period, *h*_c_ for forced convection was used to account for the effects of airflow.

Radiative (*q*_rad_) heat loss (W) was calculated for the *pre-flight*, *flight* and *post-flight* periods using the following relationship:

2.2 $$q_{\mathrm{rad}}=\sigma A\varepsilon_{h}\varepsilon_{\omega }(T_{s}^{4}-T_{a}^{4}),$$

where *σ* is the Stefan–Boltzmann constant, *A* is the surface area of the body region, *ε*_h_ represents the bird emissivity (assumed to be 0.95; [10,24], *ε*_ω_ is the wall emissivity (assumed to be 1), *T*_s_ and *T*_a_ in K.

Conductive heat loss (*q*_cond_) was calculated for the area of the feet that was in contact with the wooden perch (following [25]) during *pre-flight* and *post-flight* as follows:

2.3 $$q_{\mathrm{cond}}=\frac{k_{s}\;(T_{s}-T_{p})(1-\lambda )A_{f}}{\sqrt{\left(K_{s}\pi t\right)}\;}\overset{\infty }{\underset{n=0}{\sum }}{\left(-\lambda \right)}^{n}e^{-{\left(n+(1/2)\right)}^{2}d^{2}/(K_{s}t)},$$

where *k*_s_ is thermal conductivity of skin (0.284 W m^−1^ K^−1^, value for heel assumed; [26]), *T*_p_ is the temperature of the perch, *K*_s_ is the thermal diffusivity of skin (1.41 × 10^−7^ m^2^ s^−1^, value for heel assumed; [26]), *A*_f_ is the area of the feet in contact with the perch and *t* is the time duration spent on the perch before the thermal image was recorded. Finally, *λ* was calculated as follows:

2.4 $$\lambda =\left(\frac{k_{s}}{\sqrt{K_{s}}}-\frac{k_{p}}{\sqrt{K_{p}}}\right)÷\left(\frac{k_{s}}{\sqrt{K_{s}}}+\frac{k_{p}}{\sqrt{K_{p}}}\right),$$

where *k*_p_ is thermal conductivity of the perch (0.093 W m^−1^ K^−1^, value for softwood assumed; [27]) and *K*_p_ corresponds to the thermal diffusivity of ground surface (1.44 × 10^−7^ m^2^ s^−1^, value for softwood assumed; [27]).

Evaporative heat losses could not be quantified with the current experimental set-up; however, the magnitude of these losses has previously been shown to represent only approximately 7–12% of the total heat dissipation (e.g. 7% in herring gulls (*Larus argentatus*) at 25°C [28]; 11.6% in European starlings [15]).

The sum of convective, radiative and conductive heat loss (representing an estimated 88.4% of the total heat lost), will be called hereafter *non-evaporative heat dissipation*. Measurements of surface temperature were made unilaterally and were assumed to be bilaterally identical. When calculating regional and whole-body non-evaporative heat dissipation, unilateral surface areas were doubled to account for the two sides of the body.

### 2.1. Statistical analysis

To describe the mean *T*_s_ of the three common body areas (i.e. *head*, *trunk* and *legs*) measured during *pre-flight*, *flight* and *post-flight*, we used a general linear mixed model (GLMM) including *T*_a_, period (i.e. *pre-flight*, *flight* and *post-flight*), and timing within the period (min) nested in period as independent variables and bird ID as random effect. Significance between periods was assessed using a Tukey's honestly significant difference (HSD) *post hoc* test. The same GLMM was used to describe *wing* surface temperature but without the period factor and interaction as *wing* was only measured during *flight*. To study the effects of flying condition on *T*_s_ during *post-flight*, we used a GLMM including *T*_a_, timing in *post-flight* (min), flying speed as binomial factor and flight duration (min) as independent variables and bird ID as random effect. To investigate the non-evaporative heat dissipation of each body area, we used the same GLMM to describe the mean *T*_s_ and then compare the effect of mean *T*_s_ on the non-evaporative heat dissipation during the three periods. We performed the statistical analysis using JMP v. 13 (SAS Institute Inc., Cary, North Carolina, USA). Finally, we compared the total non-evaporative heat dissipation during the three periods using ANOVA. Results are reported as means ± s.e.

## 3. Results

### 3.1. Surface temperature

**Figure 2.**
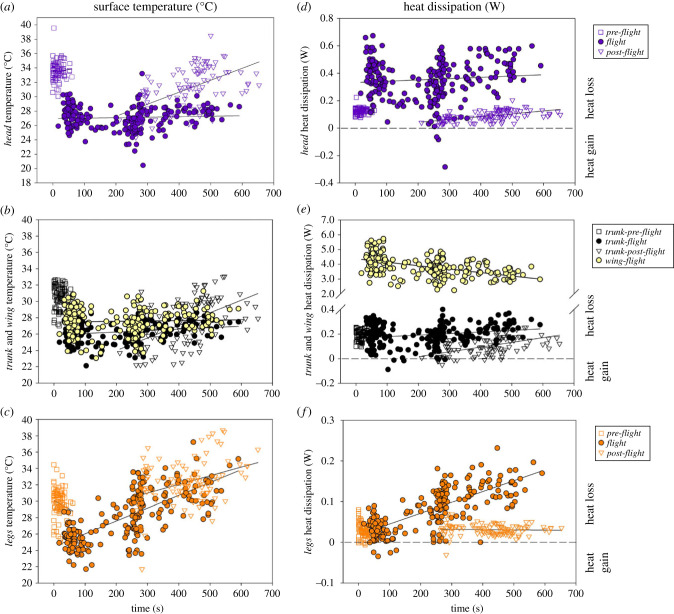
Surface temperature (*a–c*) and heat dissipation (*d–f*) of body areas: *head* (*a*,*d*) *trunk* and *wing* (*b*,*e*) and *legs* (*c*,*f*) measured in *pre-flight* (empty square) in *flight* (full circle) and in *post-flight* (empty triangle). The dashed line denotes no exchange with environment, whereas negative values correspond to heat gain and positive value to heat lost. Each dot corresponds to one thermal image and several images have been analysed per flight per period (see text for statistical details). Note that the *flight* data and the *post-flight* data have each been aligned to the same start time (because the duration of the *pre-flight* and *flight* varied).

The mean *T*_s_ of the *head* (*T*_head_), *trunk* (*T*_trunk_) and *legs* (*T*_legs_) areas were positively related to *T*_a_ (table 1) and were related to the interaction between category and time within period (table 1). Specifically, *T*_head_ remained stable in *pre-flight* as well as during *flight* but significantly increased in *post-flight* (table 1; figure 2*a*). Similarly, *T*_trunk_ remained stable in *pre-flight*, slightly increased during *flight* and increased in *post-flight* (figure 2*b*). *T*_legs_ remained stable in *pre-flight*, and significantly increased during *flight* and in *post-flight* (figure 2*c*). Finally, we measured a higher mean *T*_head_ in *pre-* and in *post-flight* (which were not significantly different from each other (*p* = 0.9780)) than in flying birds (*p* < 0.0001; figure 2*a*). Similarly, mean *T*_trunk_ was also higher in *pre-* and in *post-flight* (which are not significantly different from each other (*p* = 0.9314)) than in flying birds (*p* = 0.0003 and *p* < 0.0001 respectively; figure 2*b*). Mean *T*_legs_ was higher in *post-flight* (compared with in *pre-flight* (*p* = 0.0005) and in *flight* (*p* < 0.0001)) but was similar in *pre-flight* and in *flight* (*p* = 0.9781) (figure 2*c*). Finally, the mean *T*_s_ of the *wing* during *flight* was positively correlated to *T*_a_ (estimate of 0.76 ± 0.24; *p* = 0.0020) but remained stable throughout the flying period (*p* = 0.7092) (*n* = 224 *N* = 7, *R*^2^ = 0.14).

**Table 1.** Summary of general linear mixed models (GLMMs) investigating the determinants of *head*, *trunk* and *legs* surface temperatures. Values not sharing a common letter are significantly different for *p* < 0.05 using Tukey's HSD *post hoc* test. Bold values denote statistical significance at the *p* < 0.05 level.

	*head* (°C)					*trunk* (°C)					*legs* (°C)				
		*n* = 403	*R*^2^ = 0.76				*n* = 419	*R*^2^ = 0.69				*n* = 404	*R*^2^ = 0.62		
	*V*_residual_ = 2.63 (*n* = 403 pictures), *V*_ID_ = 0.13 (*N* = 7 individuals)					*V*_residual_ = 1.90 (*n* = 419 pictures), *V*_ID_ = 0.31 (*N* = 7 individuals)					*V*_residual_ = 4.59 (*n* = 404 pictures), *V*_ID_ = 0.64 (*N* = 7 individuals)				
	d.f.	*F*	prob. > *F*			d.f.	*F*	prob. > *F*			d.f.	*F*	prob. > *F*		
***T***_**a**_	1,394	29.96	**<0.0001**			1,409	14.70	**0.0001**			1,393	5.07	**0.0249**		
**Period**	2,395	185.93	**<0.0001**	estimates (°C)	*post-hoc*	2,409	409.00	**<0.0001**	estimates (°C)	*post-hoc*	2,394	119.58	**<0.0001**	estimates (°C)	*post-hoc*
*pre-flight*				33.67 ± 1.30	a				30.31 ± 1.03	a				27.35 ± 1.49	b
*flight*				27.26 ± 0.17	b				26.36 ± 0.24	b				27.01 ± 0.35	b
*post-flight*				33.50 ± 0.34	a				29.93 ± 0.33	a				33.30 ± 0.49	a
**Timing[period]**	3,394	45.01	**<0.0001**	estimates (°C min^−1^)		3,409	408.70	**<0.0001**	estimates (°C min^−1^)		3,393	82.82	**<0.0001**	estimates (°C min^−1^)	
*pre-flight*			*p* = 0.9187	-				*p* = 0.9187					*p* = 0.2203	-	
*flight*			*p* = 0.5580	-				*p* = **0.0112**	0.10 ± 0.04				*p* < **0.0001**	0.91 ± 0.06	
*post-flight*			*p* < **0.0001**	2.51 ± 0.22				*p* < **0.0001**	2.53 ± 0.18				*p* < **0.0001**	1.46 ± 0.27	

*T*_s_ measured in *post-flight* was positively correlated to *T*_a_ and flight duration, independently to the HDA considered (table 2). Indeed, we measured a significant but slight increase in *T*_s_ in *post-flight* as a function of flight duration in *head* (estimate of 0.009 ± 0.003°C), *trunk* (estimate of 0.008 ± 0.002°C) and in *legs* (estimate of 0.01 ± 0.003°C) (table 2), with *T*_s_ increasing with increasing flight duration. However, flight speed during *flight* only impacted the *T*_legs_ in *post-flight* (table 2).

**Table 2.** Summary of general linear mixed models (GLMMs) investigating the effect of flying condition on mean surface temperature in *post-flight* in *head*, *trunk* and *legs.* Bold values denote statistical significance at the *p* < 0.05 level.

in *post-flight*:	*head* (°C)			*trunk* (°C)			*legs* (°C)		
	*n* = 104		*R*^2^ = 0.46	*n* = 114		*R*^2^ = 0.67	*n* = 105		*R*^2^ = 0.48
	*V*_residual_ = 4.9 (*n* = 104 pictures), *V*_ID_ = 0.06 (*N* = 7 individuals)			*V*_residual_ = 2.64 (*n* = 114 pictures), *V*_ID_ = 0.78 (*N* = 7 individuals)			*V*_residual_ = 4.09 (*n* = 105 pictures), *V*_ID_ = 2.20 (*N* = 7 individuals)		
	d.f.	*F*	prob. > *F*	d.f.	*F*	prob. > *F*	d.f.	*F*	prob. > *F*
*T*_a_	1,98	23.77	**<0.0001**	1,105	16.31	**0.0001**	1,94	10.67	**0.0015**
period	1,1	17.46	**<0.0001**	1,106	106.44	<**0.0001**	1,96	25.34	**<0.0001**
speed			n.s.	1,106	3.61	0.0603	1,95	9.38	**0.0029**
speed^2^			n.s.	1,106	2.84	0.0949	1,95	6.95	**0.0098**
flight duration	1,1	14.17	**0.0003**	1,108	10.26	**0.0019**	1,95	9.50	**0.0027**

### 3.2. Mechanism of heat dissipation

**Figure 3.**
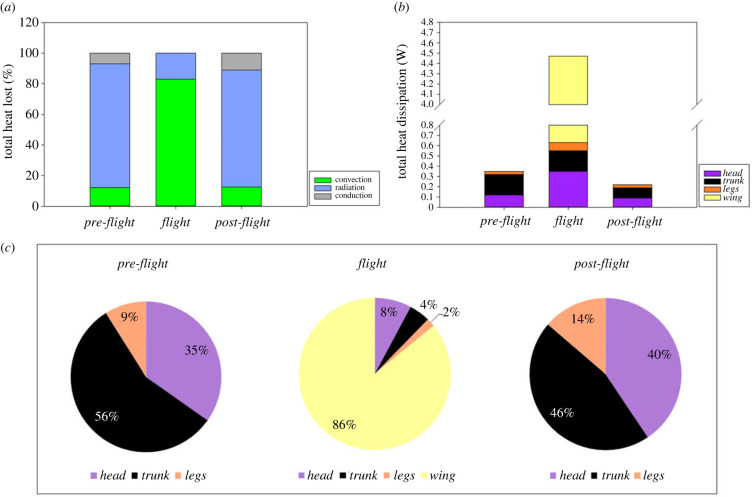
Comparison of the per cent of the total heat dissipation (%) lost by convection, radiation and conduction (*a*), the amount of heat (W) dissipated by the *head*, *trunk* and *wing* in function of experimental periods (*b*) and the representing per cent of the total heat dissipation (%) dissipated by the three body areas in *pre-* and *post-flight* and in four body areas during *flight* (*c*).

Heat was mainly lost by radiation during *pre-* and *post-flight* with a mean of 80.73% and 76.42% of the total non-evaporative heat dissipation, respectively (figure 3*a*). However, during *flight* birds lost only 17.16% of the total non-evaporative heat dissipation by radiation and the remaining 82.84% by convection (figure 3*a*). Conductive heat lost represented 6.98% and 11.10% of the total non-evaporative heat dissipation during *pre-* and *post-flight*, respectively.

### 3.3. Non-evaporative heat dissipation

The total non-evaporative heat dissipation was significantly higher during *flight* (4.46 ± 0.05 W) than in *pre-flight* (0.36 ± 0.01 W) and in *post-flight* (0.23 ± 0.01 W; paired *t*-test *p* < 0.0001; figure 3*b*) with similar heat dissipation in *pre-* and *post-flight* (paired *t*-test *p* = 0.1831; figure 3*b*). In perched individuals during *pre-flight*, the total amount of heat was mainly dissipated by *trunk* (56.6%) and *head* (34.7%) with the remainder being dissipated by the *legs* (8.7%; figure 3*c*); these HDA represent, respectively, 25%, 72% and 3% of the whole-body surface area. Thus, when normalized to surface area (i.e. heat flux), the *legs* had the highest heat flux, 0.026 ± 0.004 W cm^−2^, compared with the *head* with 0.014 ± 0.001 W cm^−2^ (*p* = 0.0041) and *trunk* with 0.008 ± 0.001 W cm^−2^ (*p* < 0.0001), which both had similar heat flux (*p* = 0.0652). In *pre-flight*, heat flux was 1.85- and 3.25-fold higher in *legs* than in *head* and in *trunk*, respectively. During *flight*, the *wing* dissipated 85.9% of the total amount of heat (figure 3*c*), but represented only 26% (i.e. ventral proximal area) of the whole-body surface area. In comparison, the *head* dissipated 7.8% of the heat, with the area representing 18% of the whole-body surface, the *trunk* dissipated 4.5% of the heat and represented 54% of the body surface, and the *legs* dissipated 1.8% of the heat and represented 2% of the whole-body surface area (figure 3*c*). During *flight*, when normalized to surface area, the *wing* had the highest heat flux (0.32 ± 0.02 W cm^−2^) compared with the *legs* (0.062 ± 0.08 W cm^−2^; *p* < 0.0001), *head* (0.042 ± 0.005 W cm^−2^; *p* < 0.0001) and *trunk* (0.008 ± 0.001 cm^−2^; *p* < 0.0001), with the *legs* exhibiting a higher heat flux than the *trunk* (*p* < 0.0001) and *head* (*p* = 0.0541), and the *head* a higher heat flux than the *trunk* (*p* = 0.0027). During *flight*, heat flux was 5.16-, 7.62- and 40-fold higher in *wing* than in *legs*, *head* and in *trunk*, respectively. In *post-flight*, when normalized to surface area, perched birds dissipated heat mainly by the *trunk* (45.7%) and *head* (40.6%), whereas the *legs* dissipated 13.7% of the total amount of heat (figure 3*c*). During *post-flight*, when normalized to surface area, *legs* had the highest heat flux 0.03 ± 0.007 W cm^−2^ compared with the *trunk* with 0.003 ± 0.02 W cm^−2^ (*p* < 0.0001) and *head* with 0.009 ± 0.004 cm^−2^ (*p* < 0.0001), and *head* exhibiting a higher heat flux than the *trunk* (*p* = 0.0135). In *post-flight*, heat flux was 10- and 3.33-fold higher in *leg*s than in *trunk* and *head*, respectively. In the three common HDA measured at each period, non-evaporative heat dissipation varied differently as a function of period and time within the period (table 3). The rate of change of non-evaporative heat dissipation by the *head* was constant in *pre-flight* and in *flight* but increased in *post-flight* (table 3, figure 2*d*). Heat dissipated by the *trunk* remained stable in *pre-flight*, slightly increased during *flight* and increased in *post-flight* (table 3, figure 2*e*). Finally, heat dissipated by the *legs* decreased in *pre-flight* increased during *flight* (figure 2*f*) and remained stable in *post-flight* (table 3). Moreover, independently of *T*_a_ (*p* = 0.3346) heat dissipated by the *wing* area was negatively correlated with flying time (slope −0.15 ± 0.02 W min^−1^; *n* = 224 *N* = 7, *R*^2^ = 0.40).

**Table 3.** Summary of general linear mixed models (GLMMs) investigating the determinants of *head*, *trunk* and *legs* dissipation*.* Values not sharing a common letter are significantly different for *p* < 0.05 using Tukey's HSD *post hoc* test. Bold values denote statistical significance at the *p* < 0.05 level.

	*head* dissipation (W)					*trunk* dissipation (W)					*legs* dissipation (W)				
	*n* = 403	*R*^2^ = 0.57				*n* = 396	*R*^2^ = 0.41				*n* = 405	*R*^2^ = 0.70			
	*V*_residual_ = 0.01 (*n* = 403 pictures), *V*_ID_ = 0.001 (*N* = 7 individuals)					*V*_residual_ = 0.005 (*n* = 396 pictures), *V*_ID_ = 0.0005 (*N* = 7 individuals)					*V*_residual_ = 0.0009 (*n* = 405 pictures), *V*_ID_ = 0.0001 (*N* = 7 individuals)				
	d.f.	*F*	prob. > *F*			d.f.	*F*	prob. > *F*			d.f.	*F*	prob. > *F*		
***T***_**a**_	1,393	0.79	0.3752			1,386	3.35	0.0681			1,394	0.84	0.3600		
**Period**	2,394	48.21	**<0.0001**	estimates (W)	*post-hoc*	2,387	0.09	0.9172	estimates (W)	*post-hoc*	2,395	33.87	< 0.0001	estimates (W)	*post-hoc*
*pre-flight*				0.08 ± 0.09	b					-				−0.03 ± 0.02	c
*flight*				0.35 ± 0.01	a				0.18 ± 0.03	-				0.06 ± 0.005	a
*post-flight*				0.13 ± 0.03	b					-				0.02 ± 0.006	b
**Timing[period]**	3,394	3.25	**0.0219**	estimates (W min^−1^)	*post-hoc*	3,386	27.93	**<0.0001**	estimates (W min^−1^)		3,395	154.56	**<0.0001**	estimates (W min^−1^)	
*pre-flight*			*p* = 0.6987	-				*p* = 0.6845	-				*p* = **0.0072**	−0.03 ± 0.002	
*flight*			*p* = 0.1452	-				*p* **< 0.0001**	0.008 ± 0.002				*p* < **0.0001**	0.02 ± 0.00	
*post-flight*			*p* = **0.0066**	0.04 ± 0.01				*p* = **0.0072**	0.07 ± 0.01				*p* = 0.6790	-	

**Figure 4.**
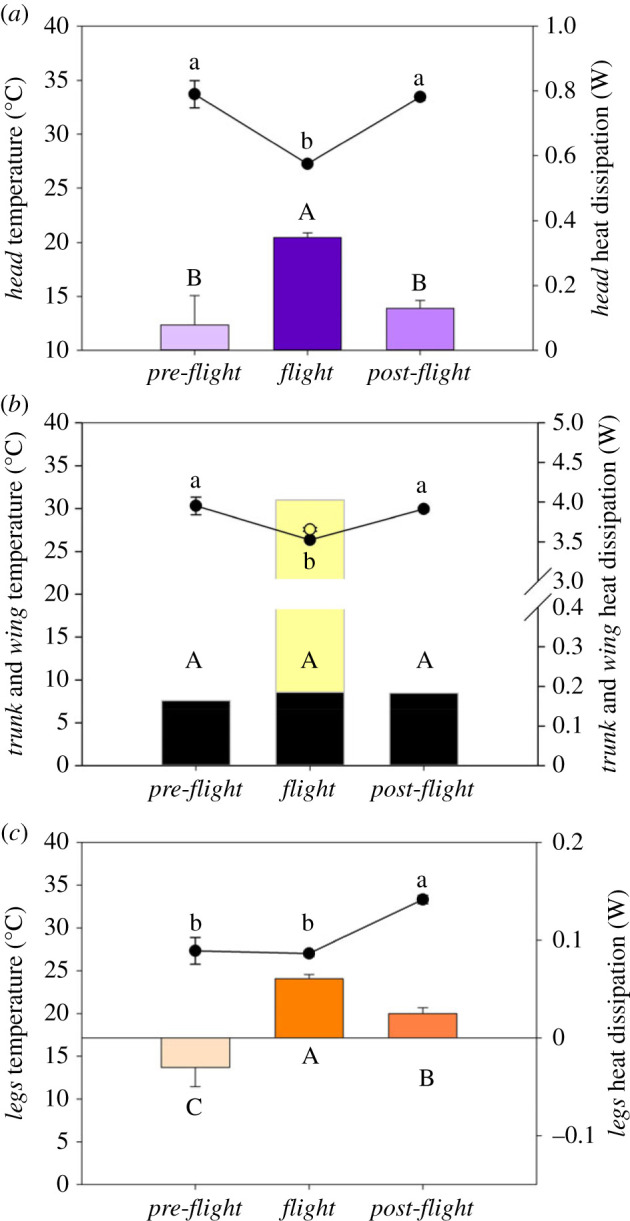
Least-square means ± s.e. are presented from the final statistical model of surface temperature (circles—table 2) and non-evaporative heat dissipation (columns—table 4) analysis in *pre-flight*, *flight* and *post-flight* in *head* (*a*), in *trunk* (*b*; yellow bar indicates heat dissipation by *wing* area during *flight*) and in *legs* (*c*). During *flight*, the filled and open circles are the surface temperature of the *trunk* and *wing*, respectively. Different letters underline a significant difference according to Tukey's HSD *post hoc* test.

Globally, the *head* dissipated more heat during *flight* than during any other periods (*p* < 0.0001; table 3, figure 4*a*). The *trunk* dissipated the same amount of heat during the three periods (table 3, figure 4*b*) and the *legs* dissipated the most of heat during *flight* (*p* < 0.0001 and *p* < 0.0001 compared with *pre-* and *post-flight*, respectively), and more heat in *post-flight* than in *pre-flight* (*p* = 0.017) with a slight heat gain (table 3, figure 4*c*).

**Table 4.** Summary of general linear mixed models (GLMMs) investigating the effect of flying condition on heat dissipation in *post-flight* in *head*, *trunk* and *legs.* Bold values denote statistical significance at the *p* < 0.05 level.

in *post-flight*:	*head* dissipation (W)			*trunk* dissipation (W)			*legs* dissipation (W)		
	*n* = 100	*R*^2^ = 0.64		*n* = 103	*R*^2^ = 0.64		*n* = 105	*R*^2^ = 0.39	
	*V*_residual_ = 0.001 (*n* = 100 pictures), *V*_ID_ = 0.00002 (*N* = 6 individuals)			*V*_residual_ = 0.002 (*n* = 103 pictures), *V*_ID_ = 0.001 (*N* = 6 individuals)			*V*_residual_ = 4.15 (*n* = 105 pictures), *V*_ID_ = 0.00 (*N* = 5 individuals)		
	d.f.	*F*	prob. > *F*	d.f.	*F*	prob. > *F*	d.f.	*F*	prob. > *F*
*T*_a_	1,92	8.98	**0.0035**	1,94	4.13	**0.0450**	1,96	7.38	**0.0078**
period	1,93	69.89	**<0.0001**	1,96	86.93	**<0.0001**			n.s.
speed	1,92	3.22	0.0757	1,95	2.94	0.0895	1,97	9.35	**0.0029**
speed^2^	1,92	2.98	0.0879	1,95	2.33	0.1305	1,97	6.75	*V*_residual_
flight duration	1,94	9.10	**0.0033**	1,96	10.01	0.0021	1,97	4.82	*V*_residual_

**Figure 5.**
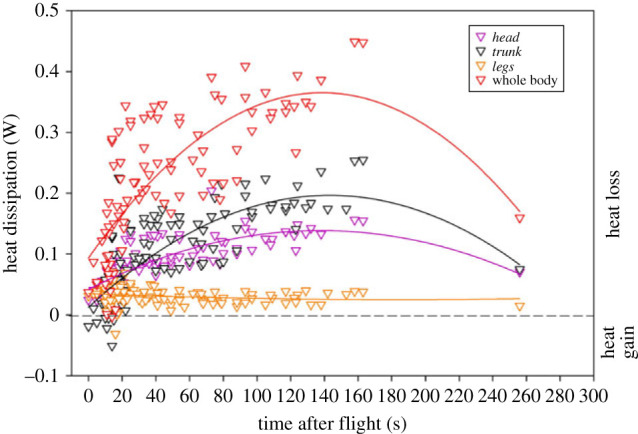
Non-evaporative heat dissipation of the *head* (purple triangles), *trunk* (grey triangles) *legs* (orange triangles) and the sum (red triangles) of these three body areas measured during *post-flight* in seven lovebirds. Positive values represent heat loss and negative ones correspond to heat gain.

Within the first 30 s after landing, the *trunk* (*n* = 8 *T*_trunk_ recorded after seven different flights led by *N* = 4 individuals) and *legs* (*n* = 1 *T*_legs_) areas both showed a net heat gain with negative heat dissipation due to the mean *T*_trunk_ (23.00 ± 0.20°C) and *T*_legs_ (21.70°C) being lower than the mean *T*_a_ (23.78 ± 0.13°C; figure 5). Based on a measurement in one bird after it had been sitting on the perch for 256 s during the *post-flight* period, it appears that eventually the rate of heat dissipation starts to decrease sometime after approximately 150 s for both the *head* and *trunk* regions as well as for the whole-body (significant binomial relationship in *head*, *trunk* and whole-body non-evaporative heat dissipation (*p* < 0.0001) but not in *feet* dissipation (*p* = 0.117; figure 5)).

Non-evaporative heat dissipation measured in *post-flight* in the three HDA was positively correlated with *T*_a_ (table 4). Moreover, heat dissipated was positively correlated with the timing after landing in the *head* and *trunk* (table 4). The heat dissipated by the three HDA increased with increased duration of the preceding flight (table 4). Finally, the preceding flight speed influenced the heat dissipated by the *legs* in *post-flight* with a slight decrease in non-evaporative heat dissipation when the preceding flight was at low speed (below 7 m s^−1^; slope −0.01 ± 0.004 W m s^−1^) but increased slightly when the preceding flight was at high speed (above 7 m s^−1^; 0.001 ± 0.00 W m s^−1^).

## 4. Discussion

Quantification of regional surface temperature has given us insight into the dissipation of heat across different regions of the body in lovebirds, before, during and after flight. According to our hypothesis, at the whole-body scale we demonstrated that total non-evaporative heat dissipation during *flight* was 12× higher than in *pre-flight* and 19× higher than in *post-flight* (figure 3*b*). The highest rate of non-evaporative heat dissipation occurred during *flight*, simultaneously lowering *T*_head_, *T*_trunk_ and *T*_legs_ compared with in the *pre*- and *post-flight* periods (figure 2*a–c*). During *flight*, convection, facilitated by the constant airflow over the bird when flying (i.e. forced convection; see below; figure 3*a*), was the primary mechanism for heat dissipation. Non-evaporative heat dissipation in perched birds during the *pre-flight* period (i.e. without airflow) remained stable in the *head* and *trunk* areas and slightly decreased in the *legs* area as a result of the largely stable surface temperatures (figure 2*a–c*), confirming that our birds were in resting state before flight. On the contrary, during the 150 s after the end of the flight (i.e. *post-flight*) when the bird was perching, there was a steady increase in heat dissipation in the *head* and *trunk* areas, but heat dissipation by the *legs* remained stable (figure 5). During *flight*, the *head* and *legs* dissipated more heat (0.35 and 0.08 W, respectively) than in *pre-flight*, whereas the *trunk* dissipated a similar amount of heat (0.18 W) during *pre-flight* and *flight*. However, the newly exposed ventral, proximal region of the *wings* in flying birds, while representing only 26% of the whole-body surface area, dissipated the highest, 85.9%, amount of heat (3.84 W of the total 4.47 W during *flight*; figure 3*c*) at the highest area-specific rate (0.32 W cm^−2^) of any region of the body.

The total metabolic rate during flight encompasses both the heat dissipated and the mechanical power generated by both the flight muscles and the circulatory and breathing systems. Therefore, the relative increase in metabolic rate is expected to be slightly higher than the increase in heat dissipation, approximately 13–14× resting metabolic rate (estimated using mechanical and metabolic power in cockatiels [16] and assuming evaporative heat loss represents 12% of the total metabolic rate [15]). The increase in metabolic rate during flight in other species ranges from 12× to 19× that at rest (13.8× in cockatiel *Nymphicus hollandicus*; 19.0× in budgerigars *Melopsittacus undulates* [29]; 12.0× in barnacle goose *Branta leucopsis* and bar-headed goose *Anser indicus* [30]; 13.5× in Europeans starlings *S. vulgaris* [31]), which is similar to the range estimated from the change in non-evaporative heat dissipation during *flight* in lovebirds. During *flight*, the overall non-evaporative heat dissipation by radiation and convection was 4.5 W. In a separate study on the same individuals (A Lewden, CM Bishop and GN Askew 2018, unpublished data), the gross metabolic rate measured using respirometry ranged from 7.5 to 8.2 W across the same speed range. Assuming an overall flight efficiency of 16.5% [30], the expected heat dissipation is 6.3–6.8 W, of which we estimate 88% is lost via radiation and convection (12% being lost via evaporative water loss [30]) giving 5.5–6.0 W of sensible heat loss. Our estimates from infrared thermography, 4.5 W, are a little lower than this estimate of the heat dissipated based on respirometry measurements. There are several possible reasons for this discrepancy: (i) during respirometry the bird flew while equipped with a respirometry mask and tubing, which may increase the metabolic cost of flight (and therefore heat dissipation), whereas the infrared thermography flights were performed without additional equipment; (ii) heat dissipation may have been underestimated because some areas of the body were not visible in the lateral view (e.g. the back) or because of errors introduced through the assumptions in the heat transfer model; (iii) the overall flight efficiency used to predict the sensible heat loss is uncertain given that it is based on an aerodynamic estimate of mechanical power, which has inherent uncertainty [17], and metabolic power obtained using the doubly labelled water technique, which lacks precision [30]; (iv) the assumption that evaporative water loss accounts for 12% of the dissipated heat may not be correct in lovebirds; and (v) not all heat may have been dissipated, some may have been stored.

Flapping flight is the most costly mode of locomotion in terms of the rate of metabolic energy expenditure [5], and is associated with the highest known rate of heat production among endotherms [32]. In our study, the elevation of non-evaporative heat dissipation during *flight* reflected this increased heat production [7,8]. However, the larger amount of heat dissipated was not due to a larger temperature gradient between *T*_s_ and *T*_a_ in the flying bird. On the contrary, we measured a decrease in *T*_s_ for each region, resulting in lower global *T*_s_ during *flight* compared with either *pre-* or *post-flight* (figure 2*a–c*), and a lower overall temperature gradient (*T*_s_–*T*_a_) maintained during *flight* at +3.77°C in *trunk* and at +14.04°C in the eye region in our lovebirds, which is similar to the temperature gradient measured in the trunk (5°C) and in eye region (15°C) in both hummingbirds [9] and pigeons [33]. In addition to the temperature gradient, the effectiveness with which heat is lost during flight was determined by the airflow over the bird during flight, which results in heat dissipation by forced convection. In the absence of airflow when the bird is perched, heat lost by free convection represents a mean of 12% of the total heat lost in *pre-* and *post-flight* (figure 3*a*), whereas during *flight* heat lost by forced convection rose to a mean of 83% of the total heat lost (figure 3*a*).

In *post-flight*, after the bird had landed, we measured a decrease in non-evaporative heat dissipation to a level that was 19× lower than during *flight* (figure 3). We also found that *T*_s_ was related to the preceding flying condition with a higher *T*_s_ after longer flights and at low and high flight speeds (table 2), where the mechanical power requirements are expected to be higher ([34]; e.g. in budgerigar [7,29]; e.g. in cockatiel [16,17]). This probably indicates that some heat was being stored early on in flight, with a consequent rise in body temperature. As a result, there was also a higher heat loss while perching after longer flights in the *head, trunk* and *legs* areas and at low and high flight speeds by the *legs* (table 4). This relationship supports the idea that non-evaporative heat dissipation was adjusted as a function of flight metabolic rate (e.g. flight speed) and was dependent on the body areas (e.g. higher in the unfeathered *legs* compared with the feathered areas of the *trunk*). Moreover, even with a small range of *T*_a_ variation (between 23 and 24.5°C), we measured a significant effect of *T*_a_ on *T*_s_, suggesting that environmental conditions could impact the thermoregulatory response and homeostasis. Supporting this suggestion are measurements made on wild birds. For example, at higher air temperatures pink-footed geese (*Anser brachyrhynchus*; [35]) are less likely to fly, godwits (*Limosa limosa*; [36]), great reed warblers (*Acrocephalus arundinaceus*; [37]) and great snipe (*Gallinago media*; [38]) fly at higher altitudes and hyperthermia in common eiders (*Somateria mollissima*), has been suggested to explain their use of stopovers [39].

Excess post-exercise oxygen consumption in humans following cycling increases with exercise duration and intensity [40]. The mechanisms underlying the increased heat dissipation and oxygen consumption following activity are multifactorial, and include increased ventilation, circulation and muscle temperature, as well as replenishment of muscle and liver glycogen stores, resynthesis of creatine phosphate and ATP [40]. Therefore, it is not surprising to observe that *post-flight* heat dissipation was higher than before the flight, though heat dissipation remained at a lower rate *post-flight* than during *flight* (figure 2*d–f*), primarly due to the loss of forced convective cooling via the extended wings. Surprisingly, we found that the body surface temperature was lower immediately after flight compared with immediately before flight (figure 2*a–c* and see *head* area on the electronic supplementary material, figure S1). Moreover, our results also show a switch in the importance of the main HDAs in *post-flight* compared with those involved during *flight*. These changes are related to differences in the bird's posture and activity. During *flight*, the proximal wing, which was exposed when the wing was extended during flight, dissipated heat at a rate 11× higher than that lost by the *head*, 19× higher than the heat lost by the *trunk* and 48× higher than the heat lost by the *legs*, representing 86% of the total non-evaporative heat dissipation (figure 3*c*). Whereas when the birds were perched with their wings folded, the *trunk* and *head* assumed the major sites of heat dissipation, accounting for 91% in *pre-flight* and 86% in *post-flight* of the total non-evaporative heat dissipation (figure 3*c*). However, it is interesting to note that the increase in *T*_trunk_ of +3.58°C in *post-flight* compared with that in *flight* could be mainly due to a higher *T*_s_ in the shoulder area as observed in *post-flight* (figure 1).

The similarity in *T*_legs_ between *pre-flight* and *flight*, despite the increase in airflow over the legs during *flight*, indicates that peripheral blood flow to the legs is higher during *flight* compared with *pre-flight*. The increase in *T*_legs_ *post-flight* (32.28°C), compared with *pre-flight* (29.69°C; figures 1, 2*c* and 4*c*), suggests that peripheral blood flow to the legs is higher during *post-flight* compared with *pre-flight*. In herring gulls, it has been demonstrated that blood flow to the legs increases during flight compared with at rest, and following sustained flights the feet were warm to touch [28]. Blood flowing to the legs may also be at a higher temperature during *post-flight* compared with *pre-flight* as the heat production during activity probably induces an increase in core temperature during flight compared with at rest [28]. However, the relative increase in flow is actually slightly lower than the relative increase in metabolic rate (4.5× increase in blood flow compared with 7.5× increase in metabolic rate in flight compared with rest at 20°C [28]), which is consistent with the majority of the increased cardiac output during exercise being directed to the locomotory muscles [41]. Increasing peripheral blood flow *post-flight* and during *flight* is likely to be a mechanism to increase heat dissipation by the *legs* (figure 3*c*). In herring gulls heat dissipation by the legs accounts for 37–52% of the heat dissipation at rest. This is considerably higher than the proportion of heat dissipated by the legs in lovebirds (9% *pre-flight* and 13.7% *post-flight*), which is likely to be due to the larger relative surface area of herring gull's webbed feet compared with the unwebbed feet of lovebirds. The increase in *T*_legs_ *post-flight* compared with during *flight* is likely to reflect the more effective heat dissipation that occurs during *flight* (lowering *T*_legs_), largely as a result of forced convection during *flight*. It is possible that the increased blood flow to the legs during *flight* continues into *post-flight*, resulting in the rapid increase in *T*_legs_, since airflow and forced convective heat loss over the legs has ceased. Moreover, the large range of leg temperature measured in *post-flight* (figure 2*c*) may suggest a difference in the need to dissipate heat between birds that could be related to the core temperature attained during exercise. After flight when birds were perched and in the absence of airflow, we measured a mean increase in all *T*_s_ with +6.13°C in *head*, +3.59°C in *trunk* and +6.29°C in *leg* areas (figures 2*a–c* and 4) compared with in *flight*. The highest increase measured in the *legs* confirm that legs play an important role in avian thermoregulation [42] and we measured that independently to the surface area, the *legs* in *post-flight* showed a rate of dissipation (0.03 W cm^−2^) 10x higher than the feathered *trunk* (0.003 W cm^−2^) highlighting the advantage of exposing this area for the benefit of heat exchange. However, the initial increase in non-evaporative heat dissipation immediately upon landing followed by a decrease initiated after approximately 150 s (figure 5), suggests that muscle/body temperatures, metabolite levels and fuel reserves are returning to pre-exercise levels and the need to dissipate heat is diminishing—i.e. excess post-exercise oxygen consumption is reducing. Although *T*_s_ increased *post-flight* compared with *flight* in the *trunk*, *head* and *legs*, non-evaporative heat dissipation in all regions was lower upon landing (figure 4) due to the absence of airflow and the resulting large decrease in convective heat loss (figure 3*a*).

Comparing feathered and partially feathered areas, we found that the *head* (including eye region (i.e. periorbital region) and bill hot spots; electronic supplementary material, figure S2A), dissipated heat at 1.75× faster rate than *trunk* in *pre-flight* and at 3× faster rate in *post-flight* (figure 3*c*). Hence the lovebird's bill, which accounted for only 2.1% of the whole-body surface area, played an important role dissipating 8.4% of the total *head* heat loss in *post-flight* (electronic supplementary material, figure S2) and represents 3.4% of the total non-evaporative heat dissipation in *post-flight*; this is a smaller relative heat loss than has been measured in any other species to date. For example, in toco toucans, the bill accounted for 30–50% of the whole-body surface area and was responsible for 35–60% [12] of the total heat dissipation; in tufted puffin, the bill represented 6% of the total surface area and accounted for 18% of the total non-evaporative heat loss [13]. However, the increase in *T*_head_, driven by the increase in bill and eye *T*_s_ in lovebirds (electronic supplementary material, figure S2B), contrasts with a global decrease of bill *T*_s_ measured within the first 35 min after landing in tufted puffin [13]. In addition to interspecific variation in heat dissipation mechanisms, differences in the environmental conditions and the time at which the measurements were made could also explain the discrepancy between the studies. In our experimental set-up birds were perched in still air, whereas puffins were studied in their natural habitat on the cliffs of Alaska, where it is likely that the birds were still subjected to air currents and where the air temperature was lower (11.1–13.9°C compared with 23.7°C in this study), which probably accounts for the more rapid cooling of the bill in puffins compared with lovebirds. Moreover, the timescale could explain the two different patterns. In this study, as well as in puffins [13], the relationship between *T*_s_ and time in *post-flight* was analysed using linear regression. However, during *post-flight* (figure 5) the initial increase in *T*_s_ was followed by a tendency for *T*_s_ to decrease, though this is only supported by one measure (4.15 min after landing), which does not allow us to run an alternative statistical analysis and was excluded in our previous analysis. More data after a longer recovery period in *post-flight* would allow us to study the nature of the relationship using binomial regression; we hypothesize that bill *T*_s_ would initially show an increase followed by a decrease, the latter being consistent with the low bill temperature measured in puffin 35 min after landing [13].

In this study, we analysed thermal images of the birds in a lateral view in order to standardize the orientation and exposure of the three common HDA during the three periods [22,23]. However, not all regions of the body were visible in this view. For example, the ventral surface of the proximal wing was not exposed in perched birds; therefore, any heat dissipation from this region in *pre-* and *post-flight* was ignored. We noted that in *post-flight* some birds show behaviour such as, holding their wings away from their trunk (i.e. wing drooping), which may facilitate heat dissipation; however, we were unable to quantify the heat loss resulting from these postural adjustments in *post-flight* as the *T*_s_ of this region could not be determined in a lateral view. In addition, we observed that birds sometimes panted and used gular fluttering *post-flight*, behaviours that increase heat loss by evaporative cooling in the buccal cavity [43]. In the same way, we were unable to quantify dangling feet during *flight* as has been observed in hummingbirds and herring gulls [9,28,44] due to the small set of thermal images in which this behaviour was observed, but we clearly observed a larger part of the legs (i.e. tarsus) exposed in perched birds immediately after the end of flight than later and especially after long flights.

## 5. Concluding remarks

Understanding the mechanisms through which birds dissipate heat both during and after flight is important in enabling biologists to predict how birds may be physiologically challenged as a result of global warming, which may affect their behaviour and their survival. For example, with an increase in atmospheric temperatures, longer migratory flights could be limited by a bird's heat dissipation capacity and would require longer or more frequent stopovers, flying at higher altitudes [37,38] or changing their migration routes. In future work, using the methodological framework that we have developed in this study, investigating how birds thermoregulate across a range of ambient conditions could prove to be insightful in assessing the impact of climate change on this ecologically important group of animals.

## Data Availability

The raw data are available from the Research Data Leeds Repository: https://archive.researchdata.leeds.ac.uk/1192/ [45]. Supplementary material is available online [46].
